# Development of Dermal Lidocaine Nanosuspension Formulation by the Wet Milling Method Using Experimental Design: In Vitro/In Vivo Evaluation

**DOI:** 10.1021/acsomega.4c05296

**Published:** 2024-12-18

**Authors:** Özlem Kral, Sibel Ilbasmis-Tamer, Sevtap Han, Figen Tirnaksiz

**Affiliations:** † Department of Pharmaceutical Technology, Gazi University, Ankara 06560, Turkey; ‡ Department of Pharmaceutical Technology, Ağri İbrahim Çeçen University, Agri 04100, Turkey; § Department of Pharmacology, Lokman Hekim University, Ankara 06510, Turkey

## Abstract

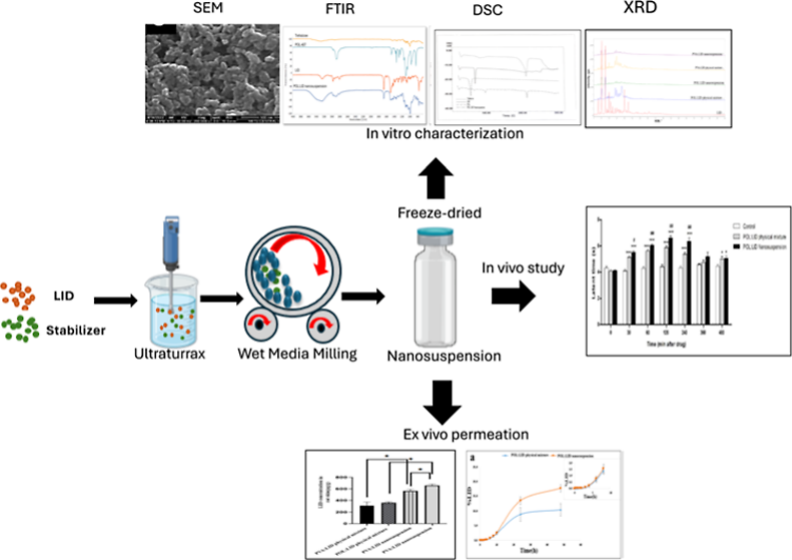

Lidocaine (LID), frequently used in dermal applications, is a nonpolar local anesthetic agent that is practically insoluble in water. The main aim of this study is to develop the nanosuspension formulation of LID using the design of experiments (DoE). The improved solubility and dissolution rate provided by nanosizing are expected to result in enhanced dermal bioavailability. Nanosuspension formulations were developed by a wet media milling method using different stabilizer types [poloxamer (POL) and poly(vinyl alcohol) (PVA)]. Characterization studies of the nanosuspensions were carried out using DSC, FTIR, XRD, and SEM in vitro release from the dialysis membrane and ex vivo permeation studies using rat skin were performed. Analgesic/anesthetic effects were evaluated using the tail-flick test in in vivo studies. Particle size (PS), polydispersity index (PDI), and zeta potential (ZP) values were found as 171.7 ± 3.52 nm, 0.251 ± 0.036, and −32.2 ± 0.907 mV for POL/LID nanosuspensions and 262.1 ± 29.42 nm, 0.453 ± 0.071, and −20.2 ± 3.50 mV for PVA/LID nanosuspensions, respectively. Compared to the coarse suspension of LID, it was determined that it accumulated in the skin approximately 1.81 times more in the POL/LID nanosuspension formulation and 1.79 times more in the PVA/LID nanosuspension formulation. According to analgesic effect and related AUC data, nanosuspension formulation was found to be statistically more effective than coarse suspension. It is concluded that DoE is a useful tool in determining process parameters when developing nanosuspensions by the wet media milling method, and POL is a suitable nonionic polymer to stabilize nanosuspensions.

## 1 Introduction

Dermal application is a way in which active ingredients can be applied effectively and efficiently through the skin.^1^ When the active substance is applied dermally, it has advantages such as providing good patient compliance, being noninvasive, creating minimal drug–drug interactions, ease of application, reducing systemic side effects in case the disease originates from the skin, and providing continuous/controlled release at the site of action.^2^ Dermal application may result in reduced pharmacological efficacy due to poor skin penetration of the active ingredients. It has been reported that various nanotechnological approaches such as liposomes, solid lipid nanoparticles, niosomes, transfersomes, ethosomes, nanostructured lipid carriers, nanoemulsions, dendrimers, and micelles can overcome these disadvantages.^3^

An alternative approach used for this purpose is to develop a nanosuspension formulation. In nanosuspensions, active substance nanocrystals are pure active substance particles smaller than 1000 nm and are stabilized with appropriate surfactants and/or polymers.^4^ The nanometer-sized stabilized particles of the active substance can be absorbed more quickly and easily through the skin and enter the underlying tissues. In nanosuspensions, active substances barely soluble in water have a large-surface area, and therefore both the dissolution rate and water solubility of the active substance increase. They provide accumulation of active substances in the skin in nanoparticulate form, increasing skin penetration and bioavailability of drug molecules by causing an increased concentration gradient.^5,6^ In addition, it has been reported that these systems increase the dermal pharmacological effect of the active substance as it accumulates in skin appendages and skin layers, especially in epidermis.^7,8^

The pH of an intact skin surface is generally 5.5, which is considered the classic cutaneous pH. This acidic pH value usually varies between 4 and 6 due to many factors such as age and gender.^9^ pH is an important parameter affecting the rate of absorption of acidic and basic drugs, and the nonionized form of the drug penetrates better through the skin. The movement of ionizable particles in aqueous solutions is largely dependent on pH.^10,11^ When the pH of the nanosuspensions is close to the pH of the stratum corneum, the nanosuspensions are in a nonionic form and the permeability of the drugs increases.^10^ For this reason, in our study, the poorly water-soluble (nonionized) base form of lidocaine was used and nanosuspension formulations were developed.

Lidocaine is a local anesthetic agent that is practically insoluble in water.^12^ In percutaneous or dermal applications, LID penetrates the stratum corneum and desensitizes pain receptors in the skin. Disadvantages such as polymorphism and low bioavailability seen in crystalline pharmaceuticals limit the transdermal application of LID.^13^ With drug carrier systems such as nanosuspensions, it is possible for an active substance to penetrate the skin more easily and to provide sustained effect with slow release of the drug substance. In recent years, targeting topically applied active substances to different skin layers as particulate carriers has become an important research topic.^14^ For this purpose, many drug carrier systems such as nanoethosomes,^15^ solid lipid nanoparticles,^14^ microemulsions,^16^ nanostructured lipid carriers,^17^ silica nanoparticles,^18^ and liposomes^19^ have been prepared containing LID. In this study, a nanosuspension formulation of LID was prepared to benefit from the advantages of nanosuspensions.

Nanosuspensions generally consist of active ingredient nanocrystals, surfactant or polymeric type stabilizers, and liquid dispersion medium.^20^ The type and amount of stabilizing agents have a significant impact on the physical stability and in vivo behavior of the nanosuspension. Examples of the most commonly used stabilizers are poloxamers, polysorbates, cellulose derivatives, povidone, and lecithin.^21^

In this study, LID nanosuspension formulations were prepared using the media milling method. This technique has advantages such as high flexibility in handling, simplicity, high reproducibility, low use of excipients, low batch-to-batch variation, and easy scale-up compared to other nanosuspension production methods.^22,23^ When preparing nanosuspension by media milling, there are many process parameters that need to be optimized such as bead size, milling time, milling speed, and bead volume.^24^ For this purpose, a factorial design with two 2^3^ (2 levels, 3 factors) three repetitions was performed separately using Design Expert software to determine the most appropriate process parameters.

The approach of using design of experiments (DOE) in quality by design (QbD) provides pharmaceutical researchers with the opportunity to obtain products in a shorter time with fewer experiments.^25^ DOE helps identify and classify (critical or noncritical) various formulation and process parameters that affect system quality. Interactions between various input variables can be detected and quantified with a well-implemented DOE. It also provides the opportunity to predict desired quality attributes over the design space.^26^ The choice of experimental design depends on the objectives of the experiment and the number of factors to be investigated.

In this study, it was aimed to develop a nanosuspension formulation that would allow LID to accumulate in the skin and have a greater anesthetic effect. The use of the base form was preferred because it penetrates the skin more easily, accumulates in the stratum corneum, and maintains its local anesthetic effect for a long time. In order to increase its dermal efficacy, LID nanosuspensions were prepared by using experimental design. The effect of process parameters on the PS, PDI, and ZP values of nanosuspensions in the wet media milling method was determined. The effect of nanosuspensions on the dermal bioavailability of LID was explained by permeation and skin accumulation experiments. Additionally, the effectiveness of the formulations was evaluated in vivo by the tail flick test.

## 2 Materials and Methods

### 2.1 Materials

Lidocaine base was kindly donated by VEM Pharmaceuticals (Turkey). POL 407 was kindly provided by BASF (Turkey), and PVA was provided by Wacker (US). All other reagents used were of analytical grade.

### 2.2 Method

#### 2.2.1 Preparation of Lidocaine Nanosuspensions

Nanosuspensions were prepared by a wet media milling method using Retsch PM100. A bead mill vessel with a volume of 50 mL was used. The coarse suspension, beads, and blank sections each worked to approximately one-third of the entire boiler.

##### 2.2.1.1 Preparation of POL/LID Nanosuspensions

0.5% POL was mixed with a magnetic stirrer until completely dissolved in distilled water. 2% LID was added to this solution and mixed with ultra turrax at 15,000 rpm for 10 min. This coarse suspension was added to the milling bowl along with the beads. The milling bowl was placed in the device, and the device was operated at the specified speed and duration. At the end of the period, the nanosuspension and beads were separated from each other using metal sieves.

##### 2.2.1.2 Preparation of PVA/LID Nanosuspensions

0.125% PVA (0.125%) was added to distilled water heated to 80 °C and mixed with a magnetic stirrer until completely dissolved. LID was added to the cooled solution, and ultra turrax was applied at 15,000 rpm for 10 min. Then, it was prepared in the same way as the process steps in the preparation of the POL/LID nanosuspensions.

##### 2.2.1.3 Preparation of Physical Mixtures

The stabilizers in the determined amounts were dissolved in distilled water, LID was added, and ultra turrax was applied for 10 min at 15,000 rpm.

### 2.3 Experimental Design for Process Parameters

To prepare nanosuspension formulations by the wet media milling method, process parameters were optimized by using experimental design. Design Expert Version 8 software was used to determine the optimal process parameters for each stabilizer. To prepare POL and PVA nanosuspensions containing PVA or POL, 2^3^ (2 levels, 3 factors) factorial designs with three repetitions were performed. The process parameters used in the prepared formulations are listed in Table 1. The process parameters of the nanosuspension were decided by the relationship between the independent and dependent variables. The independent variables were milling speed, milling time, bead size, and the dependent variables were PS, PDI, and ZP. Nanosuspensions were prepared using the process parameters in the experimental design. PS, PDI, and ZP values of nanosuspensions were measured.

**Table 1:** Dependent and Independent Variables Examined in the Experimental Design

	Polymer	POL		PVA	
		low	high	low	high
independent variables	**milling speed (rpm)**	300	400	200	300
	**milling time (hour)**	1	2	1	2
	**bead size (mm)**	0,5	1	0,5	1
dependent variables	**particle size (PS)**				
	**polydispersity index (PDI)**				
	**zeta potential (ZP)**				

### 2.4 Lyophilization of Nanosuspensions

Nanosuspensions were lyophilized using a Christ Alpha 1–2LD Freeze-Dryer. 2,5% trehalose was added to 2 mL of nanosuspension. After being frozen at −80 °C for 2 h, it was dried in a lyophilizer at −50 °C and 0.021 mbar pressure for 48 h. Then, the obtained powders were dispersed in distilled water, and PS, PDI, and ZP values were measured.

### 2.5 Characterization of Nanosuspensions

#### 2.5.1 Particle Size, Polydispersity Index, and Zeta Potential

PS, PDI, and ZP values were measured using the Malvern–Zetasizer. While measuring, 20 μL of nanosuspension was diluted to 2 mL with distilled water.

#### 2.5.2 Differential Scanning Calorimetry

DSC analysis was performed in lyophilized nanosuspensions, stabilizer-LID physical mixtures, and coarse LID powders using the DSC 60 Shimadzu instrument. Samples, weighed using a precision balance, were placed between aluminum pans and compressed. DSC thermograms between 25 and 300 °C were determined at a heating rate of 10 °C/min under nitrogen gas.

#### 2.5.3 X-ray Powder Diffraction

XRD analysis was performed on lyophilized nanosuspensions, stabilizer-LID physical mixture, and coarse LID powder with a Rigaku Ultima-IV powder diffractometer. Under 40 kV voltage, the scanning range was applied to be in the range 5–120° at a 2θ angle.

#### 2.5.4 Fourier Transform Infrared Spectroscopy

The spectra of the systems were examined by FTIR spectroscopy using lyophilized nanosuspensions. FTIR analysis was also performed with a coarse LID powder and excipients. The analysis was carried out in the scanning range of 600–4000 cm^–1^ and with the FTR-ATR disk printing technique.

#### 2.5.5 Morphological Characterization

The morphological properties of POL and PVA nanosuspensions, stabilizer-LID physical mixtures, and coarse LID powder were obtained by imaging with SEM (Quanta 400F Field Emission). Samples coated with gold–palladium were analyzed by using an acceleration voltage of 5–20 kV.

#### 2.5.6 UV Spectrophotometer Analysis of LID

The LID concentration was analyzed by an UV spectrophotometer (Cary 60, Agilent Technologies, ABD) at 265 nm wavelength in pH 7.4 phosphate buffer media. The UV method was validated for parameters such as specificity, linearity, range, accuracy, precision, and robustness.

#### 2.5.7 Determination of Drug Content

Lyophilized nanosuspensions powder was weighed (W) and dissolved in pH 7.4 phosphate buffer (V). It was mixed at 250 rpm in a magnetic stirrer, and the concentration of LID (C) was determined by an UV spectrophotometer. The drug content was calculated using eq 1(27)
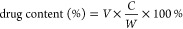
1

#### 2.5.8 Solubility Studies

Solubility studies were performed on coarse LID powder, stabilizer-LID physical mixtures, and lyophilized nanosuspensions. Distilled water or pH 7.4 phosphate buffer was taken into a glass vial, and an excessive amount of powder materials was added. It was mixed by vortex for 5 min. The samples were mixed in a water bath with a magnetic stirrer for 48 h at 37 °C. The samples were centrifuged at 15,000 rpm for 10 min, and the supernatant was separated and filtered through a 0.45 μm membrane filter and were determined with an UV spectrophotometer. The study was conducted in three parallel runs.^28,29^

#### 2.5.9 In Vitro Release Studies and Release Kinetics Studies

In vitro release studies were performed using Franz diffusion cells with dialysis membrane (cut off: 14,000 Da). pH 7.4 phosphate buffer was used as the release medium, and the study was carried out at 37 ± 0.5 °C at 500 rpm. Sink conditions were met by considering the saturation solubility of the active substances. At each time point, the concentration of the solution was adjusted to be well below the saturation solubility of the active substances. Samples were taken at specified times for 48 h, and pH 7.4 phosphate buffer was used on a release medium. The samples were filtered and analyzed with UV.

To investigate possible mechanisms of LID release from the formulations, in vitro release data were fitted to a zero-order, first-order Higuchi model, and Hixson–Crowell and Korsmeyer–Peppas kinetics using DDSolver software. The equations of the kinetic models are given in Table 2.

**Table 2:** Kinetic Model Equations Used in DDSolver to Analyze Release Data of LID^a^

model	equation
zero order	*F* = *k*_0_*t*
first order	*F* = 100 [1–Exp(−*k*_1_ *t*)]
Higuchi	*F* = *k*_H_ *t*^0.5^
Hixson–Crowell	*F* = 100 [1–(1–*k*_HC_ *t*)^3^]
Korsmeyer–Peppas	*F* = *k*_KP_ *t*^*n*^

a *F* = drug released (%); *t* = time (hours).

#### 2.5.10 Ex Vivo Permeation Study

Ex vivo skin permeation studies were carried out with the approval of the Gazi University Animal Experiments Local Ethics Committee (dated 12.02.2021 and numbered E.21975). Skin samples were taken from healthy male albino Wistar rats (230 ± 10 g) in the Experimental Animals Laboratory of Gazi University Faculty of Pharmacy. The back areas of the sacrificed rats were shaved without damaging the skin, and the entire back skin was removed. The removed skin was wiped with distilled water. The skin was then placed on the Franz diffusion cell with the stratum corneum layer facing the donor segment and the dermal layer facing the receptor segment. The formulation was placed in a donor compartment. The receptor compartment was filled with 2.5 mL of pH 7.4 phosphate buffer. Samples were taken at certain times, and pH 7.4 phosphate buffer was added instead. The samples were filtered through a 0.2 μm membrane filter and analyzed by HPLC.

As a result of the study, lag time, flux (*J*_s_), and permeability coefficient (*K*_p_) were calculated using eq 2.^30^ The lag time was determined based on the point where the linear part of the curve intersects the *x*-axis.^31^
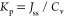
2

*K*_p_: permeability coefficient *J*_ss_: steady-state flux *C*_v_: total donor concentration

#### 2.5.11 Determination of the Amount of LID Remaining in the Skin

After the ex vivo study, the skins were removed, and the upper parts of diffusion cells were washed with distilled water. The weighed skins were cut into small pieces. 2 mL of methanol was added to the skins taken into the tubes and left for 12 h. It was then vortexed for 60 s and centrifuged at 15,000 rpm for 10 min. The filtered supernatant was analyzed by HPLC. Based on the concentrations obtained, the amount of LID remaining on the skin was calculated by taking into account the weight of the skin samples.^32,33^

#### 2.5.12 Stability Studies

Lyophilized nanosuspensions were kept in stability cabinets at 4 ± 2 °C, 25 ± 2 °C/60 ± 5% relative humidity, and 40 ± 2 °C/75 ± 5% relative humidity. Samples were taken at certain time intervals, and their characteristic properties (PS, PDI, ZP, and drug content) were examined.

### 2.6 In Vivo Studies

#### 2.6.1 Animals

Wistar albino male rats, 10 weeks old and weighing 150–200 g, were used in in vivo studies. The animals were purchased from Kobay D.H.L. A.Ş. The rats were maintained under controlled conditions (temperature 20 ± 2 °C; humidity 55 ± 10%; 12/12 h light/dark cycle). In vivo studies were carried out at the Gazi University Faculty of Pharmacy Experimental Animals Laboratory with the approval of the Local Ethics Committee dated 22.04.2021 and 79,983, received from the Gazi University Animal Experiments Local Ethics Committee.

The animals were randomly divided into three groups, and each group consisted of six animals. The experimental groups are summarized in Table 3.

**Table 3:** Design of Animal Groups

**groups**	
**group 1**	control group (without applying the formulation)
**group 2**	POL/LID physical mixture
**group 3**	POL/LID nanosuspension

#### 2.6.2 Evaluation of Local Anesthetic Effects

To evaluate the local anesthetic effect, a tail-flick test was performed by using a tail movement measuring device (Ugo Basile, Varese, Italy). The formulations were applied topically to the tail at 16.5 mg of LID per kg. Tail flick time was determined by applying radiant heat to the dorsal surface of the rats’ tails. The cutoff value of the device was determined as 10 s to prevent tissue damage due to heat application. For each rat, reaction times were recorded at 0, 0.5, 1, 2, 4, 6, and 8 h.

% Analgesic effect calculated using eq 3.^34,35^
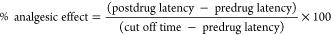
3

### 2.7 Statistical Analysis

GraphPad Prism 5.0 software was used in statistical analyses. The *T*-test was used to compare two groups, and the ANOVA test was used for further group comparisons. In the analyses, a significant difference was determined as *p* < 0.05. In vitro and in vivo studies were conducted in three parallel ways.

## 3 Results and Discussion

### 3.1 Preparation of LID Nanosuspensions

The wet media milling method is one of the important methods used in nanosuspension preparation. This technique has advantages such as simplicity, easy scale-up, and the ability to obtain narrow particle size compared to other nanosuspension production methods.^22^ Within the scope of our study, an experimental design was made while developing nanosuspension formulations. Different design types, such as factorial, Box–Behnken, and central composite, can be used for experimental design. 2^3^ factorial designs were used in our study. Factorial designs allow the experimenter to choose which factors are important and at what levels. The most commonly used (two-level design) is the full factorial design and is described as 2 k designs. Here, “2” represents the number of base levels, and *k* represents the number of high and low-value factors.^36^ The process parameters that need to be optimized when preparing nanosuspensions with the wet media milling method are generally stated in the literature as beads size, milling time, and milling speed.^24^ To determine the most suitable process parameters for each stabilizer, 2^3^ factorial designs with three repetitions (2 levels, 3 factors) were made using Design Expert software. After the formulations were prepared, PB, PDI and ZP values, which were determined as dependent variables, were measured. The compositions of the formulations prepared using different process parameters are shown in Table 4.

**Table 4:** Composition of Nanosuspension Formulations

POL–LID nanosuspensions		
Rx		
LID&.	% 2	
POL	% 0.5	
distilled water	qs	100 g
PVA–LID nanosuspensions		
Rx		
LID.	% 2	
PVA	% 0.125	
distilled water	qs	100 g

In a study, agomelatine nanosuspensions were prepared by optimizing the bead size (0.1, 0.5, and 1 mm), surfactant concentration (10, 25, and 40%), and milling speed (300, 450, and 600 rpm) parameters with an experimental design. As a result of the experimental design, it was stated that optimum nanosuspensions were prepared with 0.1 mm beads size, 10% stabilizer concentration, and 450 rpm milling speed. Nanosuspensions were prepared with PS, PDI, and ZP values of 210 ± 3 nm, 0.164 ± 0.01, and −17.2 ± 0.8, respectively.^37^

In a study using the Box–Behnken experimental design, etodolac nanosuspensions were successfully prepared by the beads milling method. Beads size (0.1, 0.5, 1 mm), milling time (1, 2.5, 4 h), and milling speed (200, 400, 600 rpm) were selected as independent variables in terms of process parameters. At the end of the study, it was stated that PS, PDI, and ZP values changed positively with 0.5 mm beads size, 400 rpm milling speed, and 2–2.5 h milling time. In this way, PS, PDI, and ZP values of nanosuspensions stabilized with PVP K30 were obtained as, 188.5 ± 1.6 nm, 0.161 ± 0.049, and 14.8 ± 0.3 mV, respectively.^38^

### 3.2 Determination of Process Parameters

A first-order model with interaction terms was chosen to fit the experimental data to determine the optimal nanosuspension. The model equation (eq 4) is
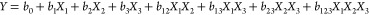
4*Y* refers to dependent variables such as PS, PDI, and ZP. *b*_0_, *b*_1_, *b*_2_, and *b*_3_ are the interaction coefficients, and *X*_1_, *X*_2_, and *X*_3_ are the coded factors for the bead size, milling time, and milling speed, respectively.

#### 3.2.1 POL/LID Nanosuspensions

**Figure 1:**
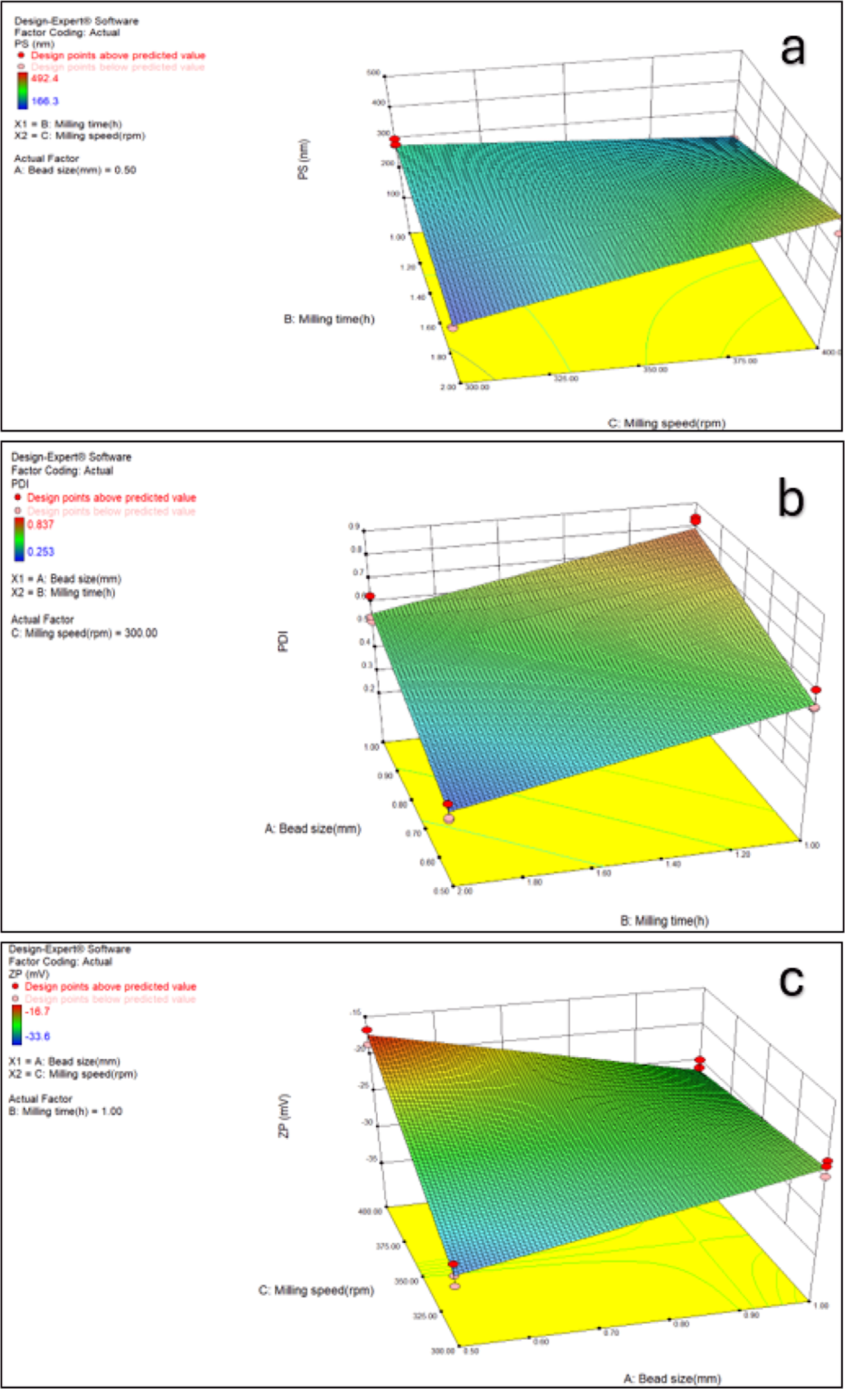
3D surface graphs demonstrating the effects of the milling time, milling speed, and bead size on PS (a), PDI (b), and ZP (c) values of POL/LID nanosuspensions.

According to the statistical analysis results of POL/LID nanosuspensions, single or dual interactions of the process parameters are significant on PS, PDI, and ZP values (Table 5). It was aimed for the particles to be nanosized (<1000 nm), PDI values to be in a narrow range (0.1–0.5) and ZP value to be ≥ ± 20 mV. When the three-dimensional graphics are examined, PS and PDI values decreased with small beads size, low milling speed, and short milling time, and ZP values were obtained close to −30 (Figure 1).

**Table 5:** Results of the Statistical Analysis for POL/LID Nanosuspensions

	PS		PDI		ZP	
	***P* value**	***F* value**	***P* value**	***F* value**	***P* value**	***F* value**
model	<0.0001	48.3	<0.0001	46.7	<0.0001	107.2
*A*-beads size	0.158	2.18	<0.0001	46.8	0.0355	5.28
*B*-milling time	0.0544	4.27	0.898	0.017	0.512	0.45
*C*-milling speed	0.138	2.42	0.327	1.02	<0.0001	444.4
*AB*	0.0231	6.23	0.475	0.53	0.0112	8.22
*AC*	<0.0001	52.8	<0.0001	45.7	<0.0001	192.8
*BC*	<0.0001	221.9	<0.0001	186.1	<0.0001	46.9
lack of fit	0.135	2.48	0.327	1.02		
*ABC*					<0.0001	52.7

As a result of the factorial design in which POL was used as a stabilizer, the model equations given below were obtained.
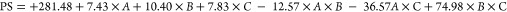

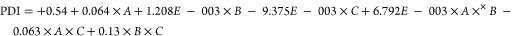

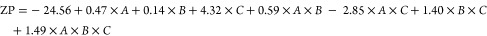
where *A* is the bead size (mm), *B* is the milling time (h), and *C* is the milling speed (rpm). *AB*, *AC*, and *BC* are the interaction between variables.

#### 3.2.2 PVA/LID Nanosuspensions

**Figure 2:**
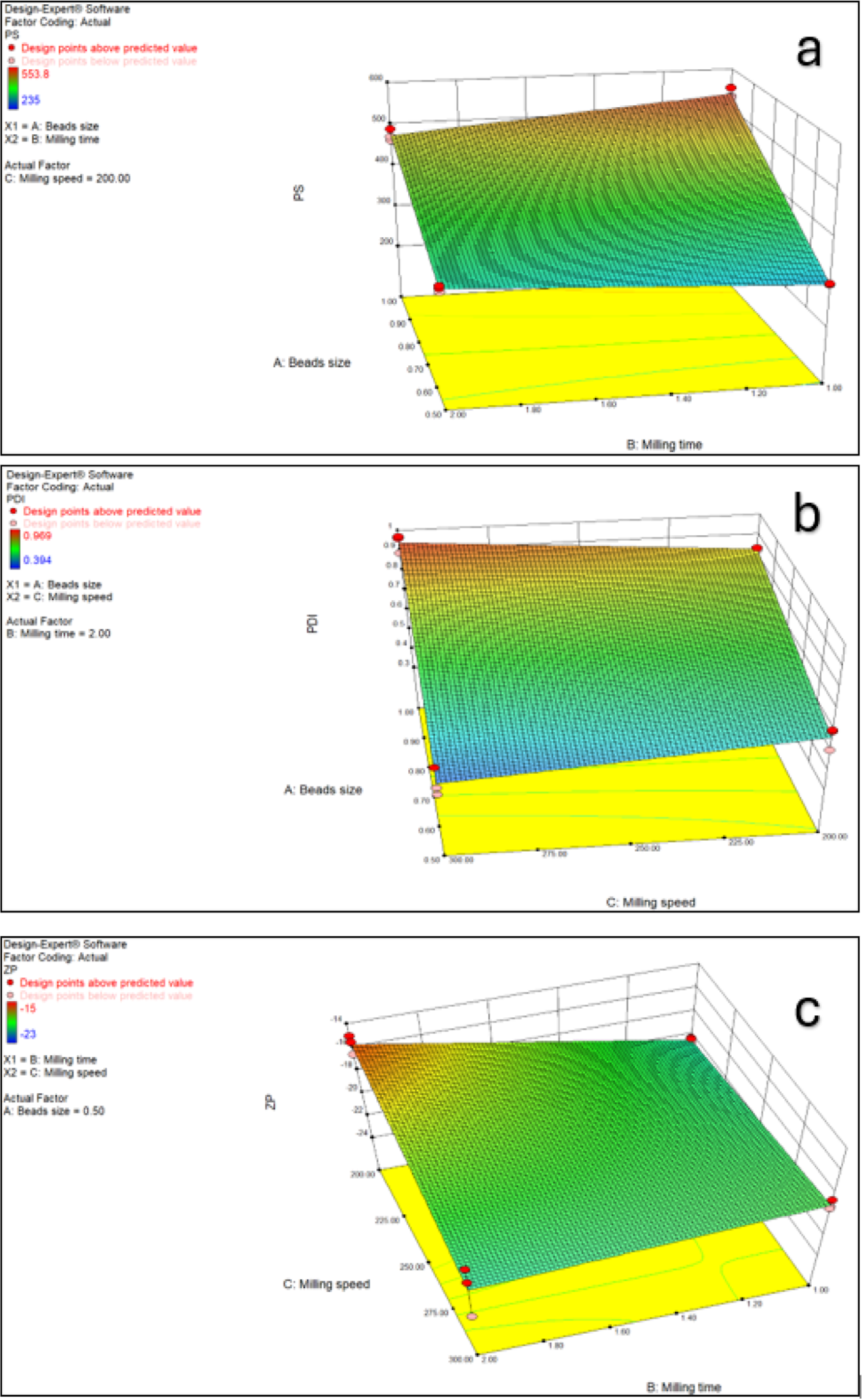
3D surface graphs demonstrating the effects of the milling time, milling speed, and bead size on PS (a), PDI (b), and ZP (c) values of PVA/LID nanosuspensions.

When the statistical analysis of the formulations stabilized with PVA was performed, all of the main process parameters and binary interactions on PS were found to be significant (Table 6). Considering Figure 2, smaller PS was obtained with high milling time, small beads size, and low milling speed. The interaction between beads size and milling time on PDI was found to be significant. When the milling speed was kept constant, nanosuspensions with narrower PDI range were produced with low beads size and long-term milling. All pairwise interactions were found to be significant on the ZP values (Table 6).

**Table 6:** Results of the Statistical Analysis for PVA/LID Nanosuspensions

	PS		PDI		ZP	
	***P* value**	***F* value**	***P* value**	***F* value**	***P* value**	***F* value**
**model**	<0.0001	111.5	<0.0001	47.1	0.0005	7.38
***A***-beads size	<0.0001	573.92	<0.0001	246.6	0.672	0.19
***B***-milling time	<0.0001	83.5	0.119	2.71	0.877	0.025
*C*-milling speed	0.0002	22.3	0.144	2.36	0.256	1.39
***AB***	0.0004	19.5	<0.0001	48.6	0.0005	18.6
***AC***	0.002	13.5	0.0584	4.15	0.0168	7.12
***BC***	<0.0001	56.3	0.109	2.88	0.0011	15.7
***ABC***	0.0037	11.6	0.0002	22.6	0.0096	8.66

As a result of the factorial design in which PVA was used as a stabilizer, the model equations given below were obtained.
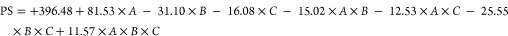

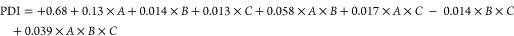

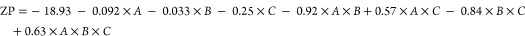
where *A* is the bead size (mm), *B* is the milling time (h), and *C* is the milling speed (rpm). *AB*, *AC*, *BC*, and *ABC* are the interaction between variables.

**Figure 3:**
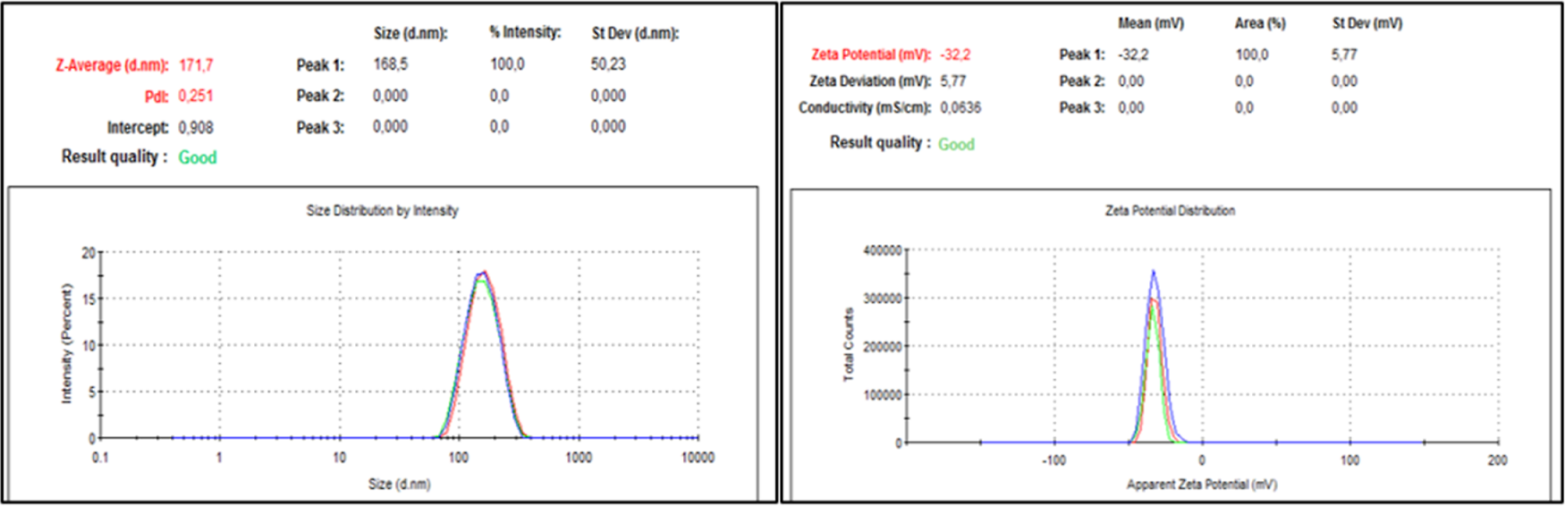
PS, PDI, and ZP values of POL/LID nanosuspensions.

**Figure 4:**
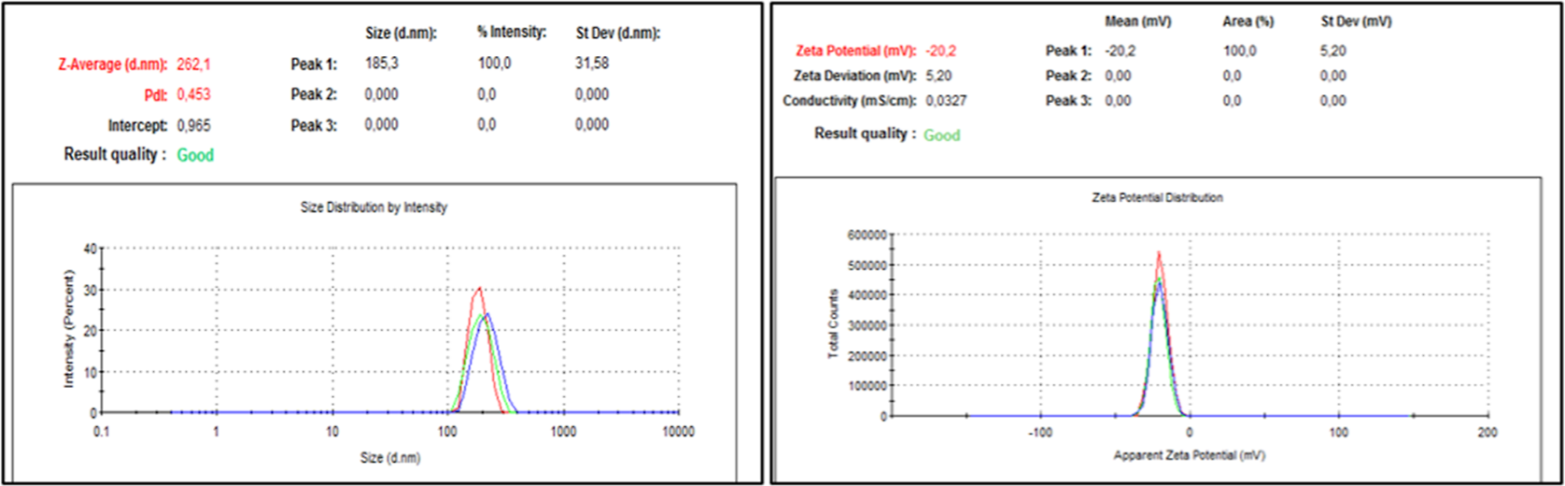
PS, PDI, and ZP values of PVA/LID nanosuspensions.

As a result of the experimental design, the most suitable process parameters for both stabilizer types were determined as 0.5 mm beads size, 300 rpm milling speed, and 2 h milling time. PS, PDI, and ZP values of nanosuspensions prepared using these process parameters are given in Table 7 and Figures 3 and 4. When the results are examined, it is seen that POL/LID nanosuspensions have smaller PS, narrower PDI, and more suitable ZP values than PVA/LID nanosuspensions.

**Table 7:** PS, PDI, and ZP Values of Nanosuspensions (X Mean ± SD)

	POL/LID nanosuspension	PVA/LID nanosuspension
**PS (nm)**	171,7 ± 3,5	262,1 ± 29,4
**PDI**	0,251 ± 0,036	0,453 ± 0,071
**ZP (mV)**	–32,2 ± 0.9	–20,2 ± 3,4

In a study, mirtazapine nanosuspensions were prepared by using different polymers and mixtures. When the active ingredient stabilizer ratio was 1:1, PS and PDI values of nanosuspensions prepared with POL were found to be 444 nm, 0.214, and nanosuspensions prepared with PVA were found to be 691 nm, 0.293, respectively. Supporting our study, better results were obtained with POL.^39^ Attari et al. prepared nanosuspensions containing different concentrations of stabilizers. It was concluded that among the prepared nanosuspensions, those stabilized with POL had lower particle size than those stabilized with PVA.^40^

Like our study, Sahu et al. (2014b) prepared nanosuspensions stabilized with PVA in their research. The zeta potential of nanoparticles was found to be negative and in the range of 5–18 mV. It has been stated that the negative charge may be due to the ionization of the carboxyl group in an aqueous environment. Additionally, the average PS of felodipine nanosuspensions was found to be 60–330 nm and the PDI to be 0.3–0.5.^41^ In another study, an olmesartan medoxomil nanosuspension using POL 407 was prepared by a combination of milling and probe sonication. Nanosuspensions were obtained with a size of 469.9 nm and exhibited negative ZP (−19.1 mV).^42^

### 3.3 Differential Scanning Calorimetry

**Figure 5:**
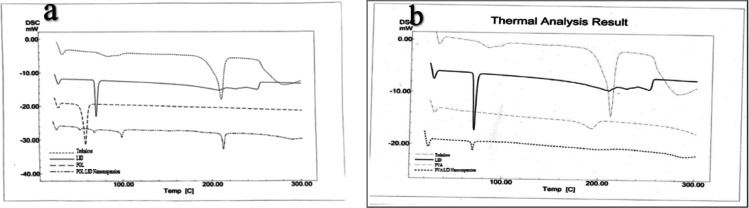
DSC thermogram of (a) POL/LID nanosuspension and (b) PVA/LID nanosuspension.

Figure 5a shows the DSC thermograms of the POL/LID nanosuspension, and Figure 5b shows the DSC thermograms of the PVA/LID nanosuspension.

DSC analysis is performed to determine whether the drug substance showed any polymorphic change or incompatibility in the prepared formulations. The melting degrees of coarse LID powder and LID in the prepared nanosuspensions were found to be similar. The structure of LID was preserved in the prepared nanosuspension formulations, and no incompatibility was observed between excipient and LID. According to the literature review, the excipients used in our study are generally utilized in nanosuspension studies and do not show incompatibility.^43,44^

### 3.4 X-ray Powder Diffraction

**Figure 6:**
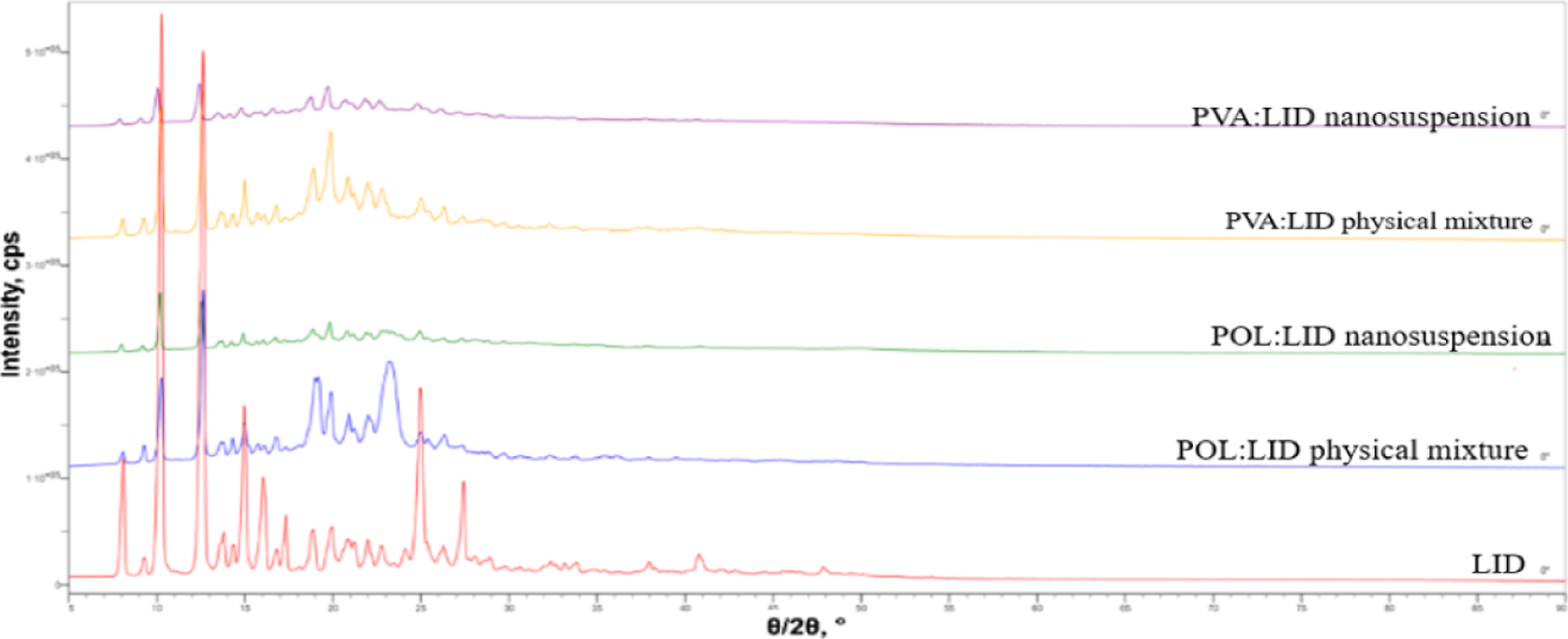
XRD patterns of coarse LID powder (LID), lyophilized POL/LID nanosuspension POL/LID physical mixture, lyophilized PVA/LID nanosuspension, and PVA/LID physical mixture.

XRD measurements are frequently encountered on nanosuspension formulations to examine the crystal structure and polymorphic change properties of the active substance.^45^ In a study, XRD measurements were made using paliperidone coarse powder, its physical mixture, and nanosuspension form. At the end of the study, characteristic peaks of the active substance are observed in the physical mixture and nanosuspension formulations. As a result, it was stated that the crystal structure of the active substance was preserved and did not undergo polymorphic change.^46^ Similarly, within the scope of our study, XRD measurements of lyophilized nanosuspensions, 1:1 physical mixtures of excipients and LID, and LID powder were made (Figure 6). It was observed that the crystal structure of the LID powder was preserved in both nanosuspension formulations. As a result, it was observed that the nanosuspension preparation method and lyophilization process did not change the crystal structure of the LID.

### 3.5 Fourier Transform Infrared Spectroscopy

**Figure 7:**
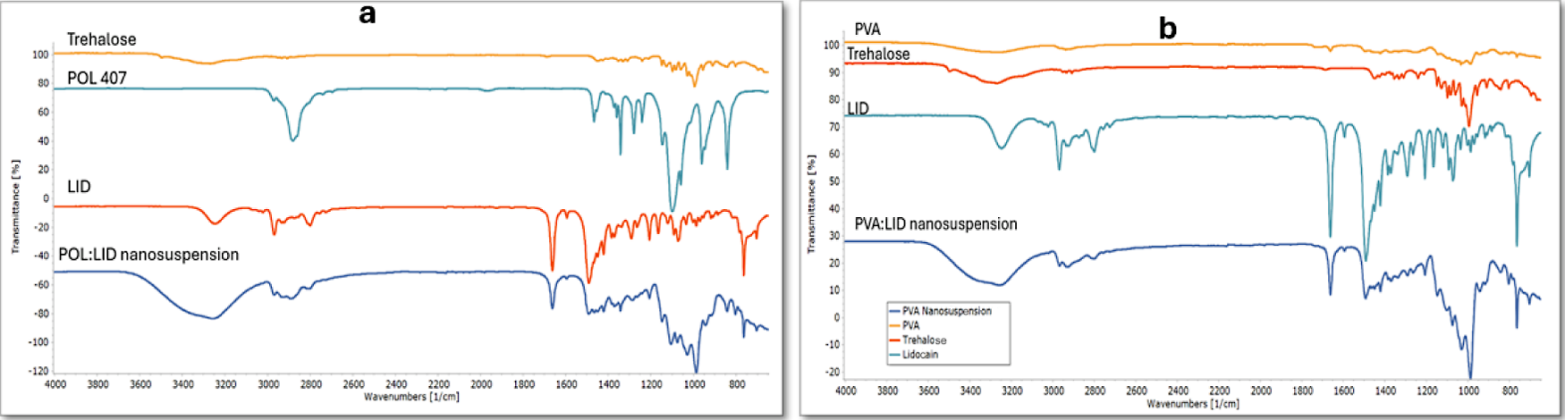
FTIR spectrum of (a) POL/LID nanosuspension and (b) PVA/LID nanosuspension.

In nanosuspension, the FTIR examination is generally performed to detect the interaction of excipients and active substances.^(^^47−49^^)^ The effect of the preparation process and the stabilizers used on the chemical structure of the nanosuspensions can be examined by FTIR analysis. FTIR spectra of lyophilized nanosuspensions prepared within the scope of this study are shown in Figure 7. The characteristics of LID are the aromatic CH stretching peak around 3000 cm^–1^, the NH bending peak around 1600 cm^–1^, the CO stretching peak around 1500 cm^–1^, and the OH bending peak around 1250 cm^–1^ in POL and PVA nanosuspensions. The results are compatible with literature information.^50^ From the FTIR results, it can be concluded that the lyophilization and wet media milling processes do not cause any effect, and the LID does not undergo polymorphic change.

### 3.6 Morphological Characterization

**Figure 8:**
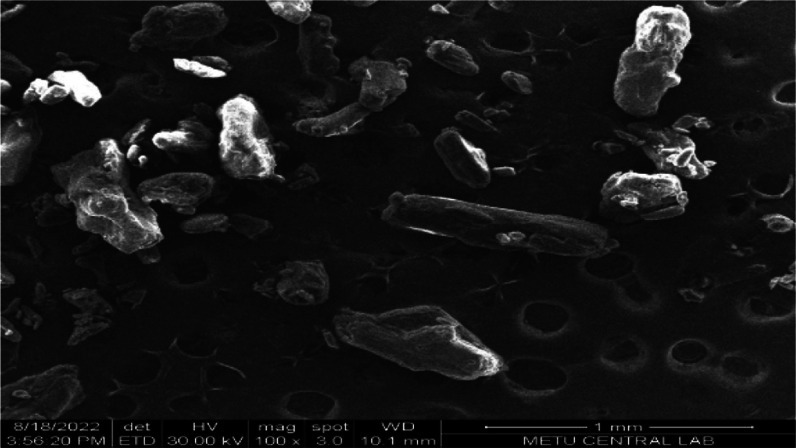
SEM image of LID coarse powder (mag.100x).

**Figure 9:**
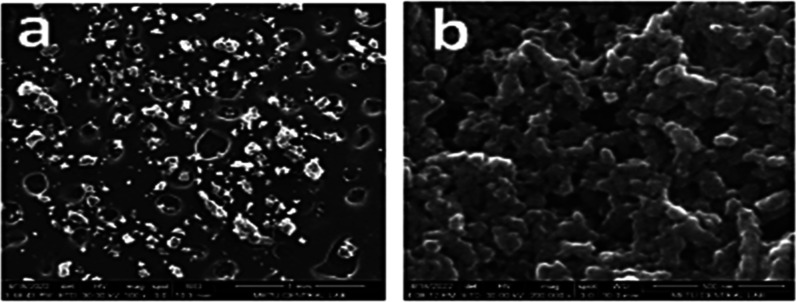
SEM image of (a) POL/LID physical mixture (mag.100x) and (b) POL/LID nanosuspension (mag.200 000x).

**Figure 10:**
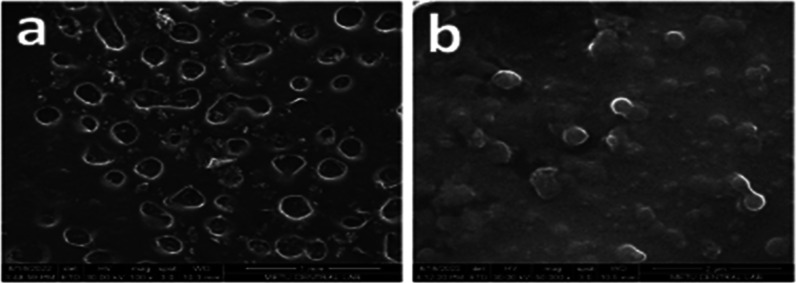
SEM image of (a) PVA/LID physical mixture (mag.100x) and (b) PVA/LID nanosuspension (mag.50 000x).

Morphological examination for nanosuspensions is an important examination that shows the shape and physical state of the particles. SEM images of nanosuspension formulations, physical mixtures, and LID coarse powder were taken to determine morphological properties. When the SEM image of the LID powder is examined in Figure 8, quite heterogeneous coarse particles are observed, similar to the literature information.^50^ Similarly, irregularly shaped active substance and stabilizer particles are seen in physical mixtures (Figures 9a and 10a). POL/LID and PVA/LID nanosuspensions were obtained in a spherical shape and nanosize (Figures 9b–10b). It has been shown in many studies that coarse powder particles and nanosuspension particles of an active substance differ morphologically as well as in size ^(^(46,47,51,52)^)^.

### 3.7 Solubility Studies

Reducing the coarse powder to a nanometer size increases the solubility of the active substance. This feature is one of the most important advantages of nanosuspensions. The results of solubility studies conducted on coarse LID powder, stabilizer-LID physical mixture, and lyophilized nanosuspensions are shown in Table 8. The solubility of lyophilized nanosuspensions was found to be higher than that of both physical mixtures and coarse powder. The solubility of the POL/LID nanosuspension was found to be 76% higher than the coarse powder and 44% higher than the physical mixture. For the PVA/LID nanosuspension, these rates were 59 and 34%, respectively. The solubility increased further with the use of POL as a stabilizer.

**Table 8:** Results of the Solubility Study (X Mean ± SD)

formulations	solubility in distilled water (mg/mL)	solubility in of pH 7.4 phosphate buffer (mg/mL)
**coarse LID powder**	3,71 ± 0,78	3,69 ± 0,57
POL/LID **physical mixture**	4,53 ± 0,50	4,47 ± 0,52
**lyophilized** POL/LID **nanosuspension**	6,53 ± 0,97	6,45 ± 0,23
PVA/LID **physical mixture**	4,42 ± 0,65	4,32 ± 0,45
**lyophilized** PVA/LID **nanosuspension**	5,93 ± 0,87	5,88 ± 0,69

Obtaining active substance particles in the nanometer range increases the saturation solubility and the dissolution rate of the drug because of the larger surface area. As a result of increased solubility, the concentration gradient between the active substance and physiological membranes increases, and the thermodynamic activity increases. This results in higher passive diffusion.^53^ There are many nanosuspension studies on this subject.^54−56^

Shah et al. (2021) conducted a comparative solubility study between lumefantrine nanosuspensions and crude active substances. At the end of the study, the saturation solubility of the rough lumefantrine powder was found to be 212.33 μg/mL, while the nanosuspension form was found to be 1670 μg/mL, a 7.8-fold increase was observed.^55^ In another study, lutein nanosuspensions were prepared for their dermal use. In the solubility study, an increase in the solubility of the prepared nanosuspensions was observed compared to lutein powder.^57^ Assem et al. (2019) conducted a comparative solubility study of beclomethasone dipropionate nanocrystals for the treatment of atopic dermatitis. As a result, it was concluded that nanosuspensions have higher solubility compared to coarse powder.^58^

### 3.8 In Vitro Release Study

**Figure 11:**
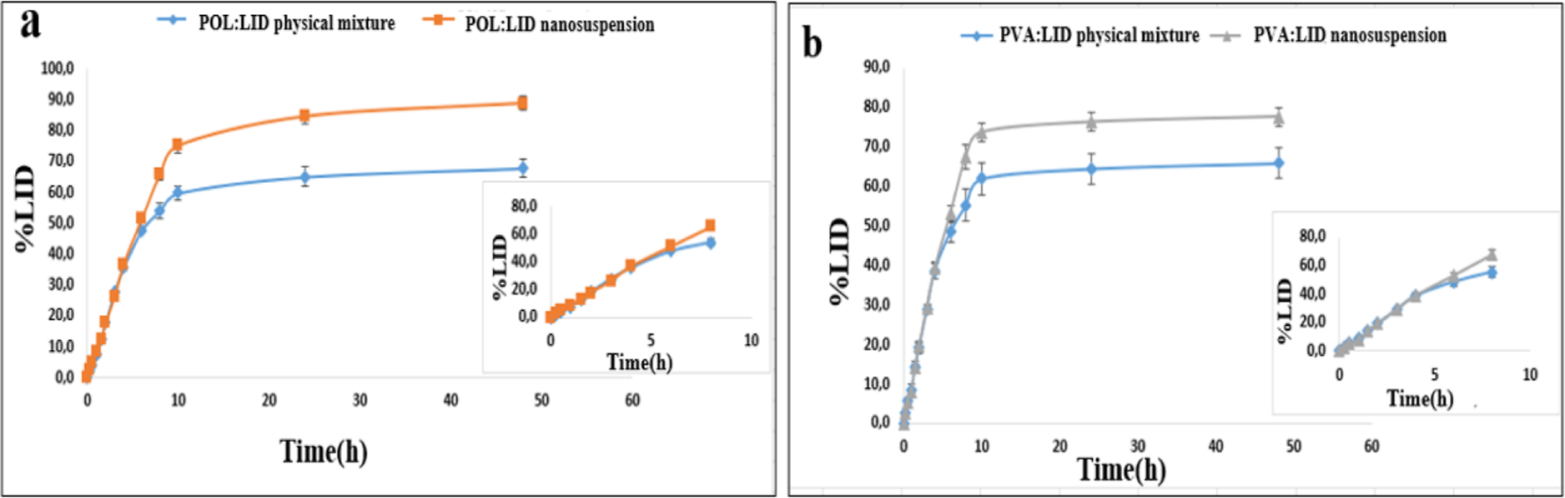
In vitro release profiles from the dialysis membrane. (a) POL/LID nanosuspension and (b) PVA/LID nanosuspension.

When the in vitro release profiles were examined (Figure 11), the amount of % LID released by POL/LID and PVA/LID nanosuspensions at 48 h was 1.3 times and 1.17 times higher than their coarse suspensions, respectively. In addition, it was determined that the % LID amount passing through the dialysis membrane at 12th, 24th, and 48th hours with POL nanosuspension was significantly higher than with PVA nanosuspension (*p* < 0.05). The difference between the in vitro release rates of coarse suspensions and nanosuspensions is due to particle size. According to the Ostwald–Freundlich formula, the solubility of active substances increases as a function of particle size.^59^ According to the Noyes–Whitney equation, as the surface area increases, the release rate/dissolution rate or the total amount of released/dissolved substance also increases.^60^

Elmowafy et al. (2021) developed luteolin nanosuspensions with antioxidant and anti-inflammatory effects using different stabilizers. As a result of the in vitro release study, nanosuspension formulations (617.3 ± 25.6 and 468.1 ± 18.6 nm) showed significantly more release than the coarse luteolin (2827 ± 27.9 nm). In addition, nanosuspensions containing different stabilizers with smaller particle sizes showed better release. It has been stated that this is due to the particle size difference, not the stabilizer.^61^ Shen et al. (2018) developed nitrofurazone nanosuspensions. In the in vitro release study, at the end of 2 h, the nanosuspension form (89.5%) showed a significant increase in the dissolution rate compared to the physical mixture (26.3%) and the active ingredient powder (23.8%).^62^ In another study, Mitri et al. (2011) prepared lutein nanosuspensions with a particle size of approximately 429 nm. In an in vitro release study using a cellulose nitrate membrane, it was determined that the prepared nanosuspensions showed higher release compared to coarse suspensions.^57^ These results support our study.

DDSolver provides a number of statistical criteria to evaluate the dissolution model. Among these parameters, *R*_2_ adjusted, AIC, and MSC are the most popular and widely used in the identification of dissolution data modeling. When evaluating these data, this means that the mathematical model is more suitable for the release profiles as *R*^2^_adj_ approaches 1, AIC decreases, and MSC increases.^63^ Statistical parameters of the models describing LID release are listed in Table 9. The values of the mathematical method fit to the release kinetics of the formulations are shown in bold.

**Table 9:** Statistical Parameters of Models to Describe In Vitro Release of LID^a^

		POL/LID physical mixture	PVA/LIDphysical mixture	POL/LID nanosuspension	PVA/LID nanosuspension
**zero order**	***R***^**2**^_**adj**_	0,945	0,933	0,990	0,976
	**AIC**	63,33	65,90	49,11	58,54
	**MSC**	2,58	2,37	4,27	3,41
**first order**	***R***^**2**^_**adj**_	**0,992**	**0,991**	0,983	0,990
	**AIC**	**42,30**	**44,3**	54,36	48,61
	**MSC**	**4,49**	**4,33**	3,79	4,31
**Higuchi**	***R***^**2**^_**adj**_	0,915	0,929	0,882	0,897
	**AIC**	68,09	66,51	75,86	74,38
	**MSC**	2,15	2,32	1,84	1,97
Hixson–Crowell	***R***^**2**^_**adj**_	0,984	0,979	**0,996**	**0,997**
	**AIC**	50,05	52,89	**39,82**	**34,61**
	**MSC**	3,79	3,56	**5,11**	**5,59**
Korsmeyer–Peppas	***R***^**2**^_**adj**_	0,937	0,955	0,994	0,984
	**AIC**	65,71	62,29	42,95	54,73
	**MSC**	2,37	2,70	4,83	3,76
	***n***	0,95	0,87	0,93	0,77

a “*R*^2^_adj_” means adjusted coefficient of determination, “AIC” means Akaike information criterion, and “MSC” means model selection criteria, “*n*” is the diffusion exponents, indicative of the drug release mechanism.

For both stabilizers, the most suitable model in the physical mixtures follows first-order kinetics. In nanosuspension formulations, release compatible with the Hixson–Crowell kinetics occurs. Chirumamilla et al. developed Meropenem nanosuspensions and conducted release studies. When the kinetic models were evaluated, as the particle size decreased, the release model switched from first-order kinetics to the Hixson Crowell model. This situation is explained as change in surface area to volume with time could be the probable reasons for increased solubility and dissolution of poorly soluble active ingredient on nanonization.^64^ Similarly, in our study, coarse forms are compatible with first-order kinetics, and nanosuspensions are compatible with Hixson–Crowell.

### 3.9 Ex Vivo Permeation Study

**Figure 12:**
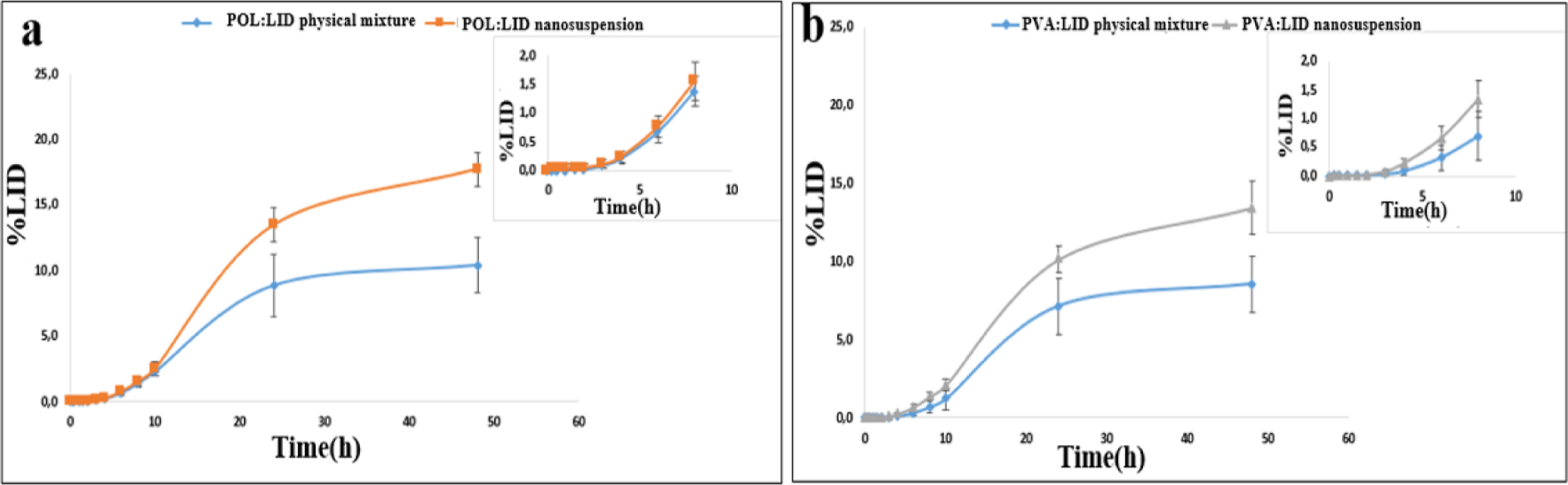
Ex vivo permeation profiles through rat skin: (a) POL/LID nanosuspension and (b) PVA/LID nanosuspension.

Skin penetration of the active substance from nanosuspensions is increased due to increased saturation solubility compared to μm-sized crystals.^65^ This causes the concentration gradient to increase between the dermal formulation and skin, subsequently leading to a higher diffusion flux. In addition, since nanomaterials are quite sticky, they increase the time they stay on the skin.^66^ In ex vivo skin permeation profiles made using rat skin (Figure 12), it was shown that the % LID amount passing through the skin at 48 h with POL and PVA nanosuspensions was 1.7 times and 1.57 times higher, respectively, than their coarse suspensions. In addition, it was determined that the % LID amount passing through the skin at 48 h with POL nanosuspension was significantly higher than that with PVA nanosuspension. This can be attributed to the particle size difference, and the penetrating power of POL 407. POL 407 is a nonionic surfactant, and it has been stated that it can interact with the skin, causing disruption of the lipid barrier in the horny layer and increasing skin permeability.^61^ Pireddu et al. compared diclofenac acid nanosuspension and coarse powder in their ex vivo study using mouse skin. The amount of diclofenac passing through the skin after 24 h was found to be higher in the nanosuspension form than that in the coarse form.^67^

As a result of ex vivo study, value of flux, permeability coefficient, and lag time were calculated (Table 10). The lag time obtained as a result of our study was found to be similar to the literature in which nanosuspension formulations were studied.^6^ When the flux values of the formulations are compared, POL/LID nanosuspensions are 1.65 times higher than coarse suspensions, and PVA/LID nanosuspensions are 1.36 times higher than coarse suspensions. The flux value of the POL/LID nanosuspension was 1.38 times higher than that of the PVA/LID nanosuspension. In addition, the permeability coefficient values of nanosuspension formulations were found to be higher than coarse suspensions.

**Table 10:** Flux, Permeability Coefficient, and Lag Time Values Calculated as a Result of Ex Vivo Permeability Study (X Mean ± SD)

formulations	flux (μg cm^–2^ h^–1^)	permeability coefficient (cm/h)	lag time (h)	*r*^2^
PVA–LID **physical mixture**	82.1 ± 17.3	0.004 ± 0.001	6.79 ± 1.04	0.997
POL–LID **physical mixture**	93.8 ± 28.9	0.005 ± 0.001	5.85 ± 0.46	0.998
PVA/LID **nanosuspension**	111.9 ± 7.3	0.006 ± 0.000	5.73 ± 0.42	0.998
POL/LID **nanosuspension**	155.2 ± 10.4	0.008 ± 0.001	5.73 ± 0.31	0.998

In a study, nanosuspension, physical mixture, and coarse suspensions of glabridin permeation from skin was compared ex vivo using Franz diffusion cells and Sprague–Dawley rat skins. As a result, glabridin permeation was higher in the nanosuspension formulation compared with the physical mixture and coarse suspension. Flux values were found to be higher in nanosuspension formulation than that in physical mixture and coarse suspension.^68^ When Romero et al. (2016) compared cyclosporin-A nanosuspensions and coarse suspensions in their ex vivo study on pig ear skin, the nanosuspension form gave better results.^69^

Applying the active ingredient in a nanoscale formulation can increase skin permeability through various mechanisms. Nanoparticulate systems have a large-surface area that increases the saturation solubility and the dissolution rate of the active substance. Additionally, they can increase diffusion by creating a high concentration gradient between the formulation and the skin.^61^ They can easily pass through the stratum corneum and penetrate the dermal sublayers through sweat glands and hair follicles. It is thought that the storage effect, which occurs through accumulation in hair follicles, is effective in the penetration of nanosuspensions through the skin.^70^ Additionally, it is important that the dermal nanosuspension has a negative ZP value. It has been reported that a negative ZP can easily propagate through the skin and diffuse into the lower layers more easily with the effect of electrostatic repulsion due to the skin being anionic.^71^ The nanosuspension formulations developed in this study have negative ZP. It has been concluded that this has a positive effect on the passage of the active substance through the skin and its accumulation in the skin.

### 3.10 Determination of the Amount of LID Remaining in the Skin

**Figure 13:**
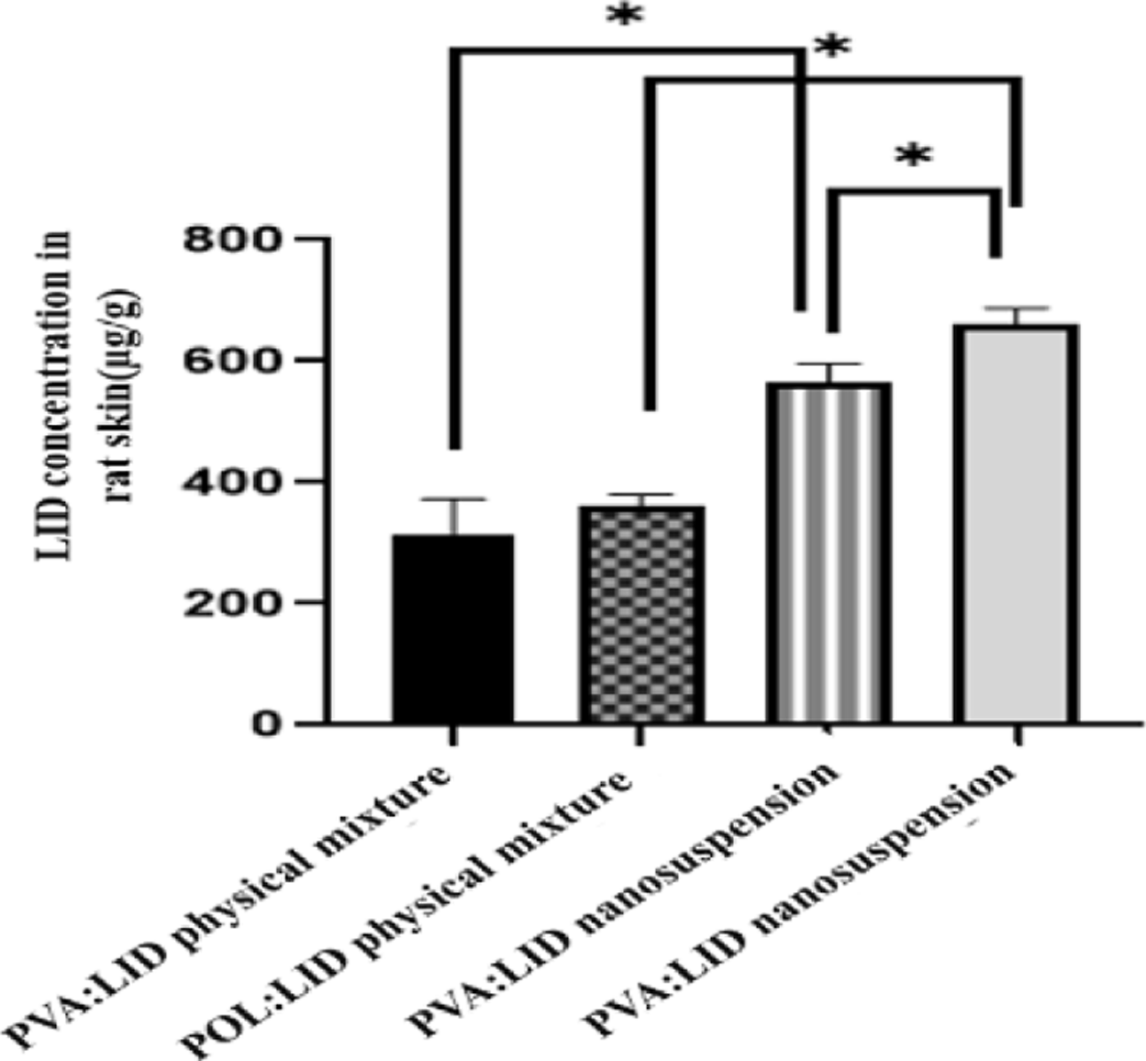
LID concentration remaining in rat skin after ex vivo permeation study (*n* = 3,*p < 0.05).

For nanosuspension formulations where local effects are expected, the aim is for the active ingredient to accumulate in the skin and not pass into systemic circulation. Therefore, studies showing that the active substance accumulates in the skin are very important. As a result of the ex vivo permeation study, the amount of LID remaining in the skin was also determined (Figure 13). The statistical analysis showed that the amount of LID remaining on the skin at the end of the 48th hour with both POL and PVA nanosuspensions was significantly different compared to the coarse suspensions. Additionally, it was determined that the amount of LID remaining on the skin at the end of the 48th hour with POL nanosuspension was significantly different from that with PVA nanosuspension (*p* < 0.05; *n* = 3).

Because of the poor solubility, after penetration of a few drug molecules in solution through a biological membrane, further dissolution of active crystals is not rapid enough to replace the penetrating molecules. Consequently, the rate-limiting step for the absorption of such drugs is the dissolution rate. In contrast to this situation, nanocrystals have increased dissolution rates due to their large-surface area and higher saturation solubility than the coarse active substance. The active ingredient molecules dissolved in the nanosuspension system, penetrated the skin causing the larger concentration gradient, and diffused in the stratum corneum by creating an accumulation area.^72^ Since nanosuspensions penetrate the skin better, they accumulate more in the skin than coarse powder. After entering the skin, a better local effect is achieved because the dissolution rate of nanosuspensions is greater than that of the coarse powder.

In their ex vivo permeation study, Manca et al. (2020) aimed to increase the accumulation of active substances in the skin by using quercetin nanosuspensions. At the end of the study, nanosuspension formulations provided more quercetin accumulation in the stratum corneum, epidermis, and dermis compared to the coarse suspension.^5^ Skin penetration of active substances in nanocrystal form increases because their size is at a level that allows them to move within the skin compared to μm-sized crystals. In addition, their high-surface area makes it easier for them to dissolve in the tissue while they diffuse through the skin.^66^

### 3.11 Determination of Drug Content

The amount of LID in the lyophilized nanosuspension prepared with both stabilizers was found to be 92.75 ± 2.35% for POL/LID and 90.57 ± 1.78% for PVA/LID. One reason why nanosuspensions are effective drug formulations is that they generally offer relatively high drug loading.^10^ In a study, a nanosuspension formulation of Rutin, one of the plant secondary metabolites with antioxidant properties, was developed using the media milling method. Drug loading capacity was found as 97.66 ± 3.33%.^73^

### 3.12 Stability Studies

The issue of stability is an inevitable problem encountered in the development of nanosuspension technology, and pharmaceutical industrial application is also the limiting step in the development of nanosuspension formulations. Nanosuspensions have a large-surface area due to their particle size and high-surface energy, causing agglomeration of the particles. Nanosuspension increases the dissolution of the active ingredient and can cause nanoparticle growth. Flocculation or nanoparticle growth during the manufacturing process or shelf life of nanosuspensions directly affects dissolution and in vivo performance due to the formation of larger particles.^74^

The main function of the stabilizer used in nanosuspensions is to obtain physical stability of formulation by surrounding the active substance particles and providing a steric or ionic barrier. This barrier prevents Ostwald ripening and aggregation of the nanoparticles.^21^ In our study, while preparing nanosuspensions, POL and PVA were used as stabilizers based on preliminary studies. POL is approved by the FDA as a pharmaceutical ingredient and is one of the most widely used hydrophilic nonionic surfactants. Additionally, it has been widely studied because of surrounding the surface of nanocrystals.^75^ PVA is a well-established excipient used in various biomedical and pharmaceutical products due to its nontoxicity, noncarcinogenicity, and bioadhesion properties. PVA also acts as a good stabilizer for nanosuspensions, increasing the system’s stability by providing a steric barrier.^76^

**Figure 14:**
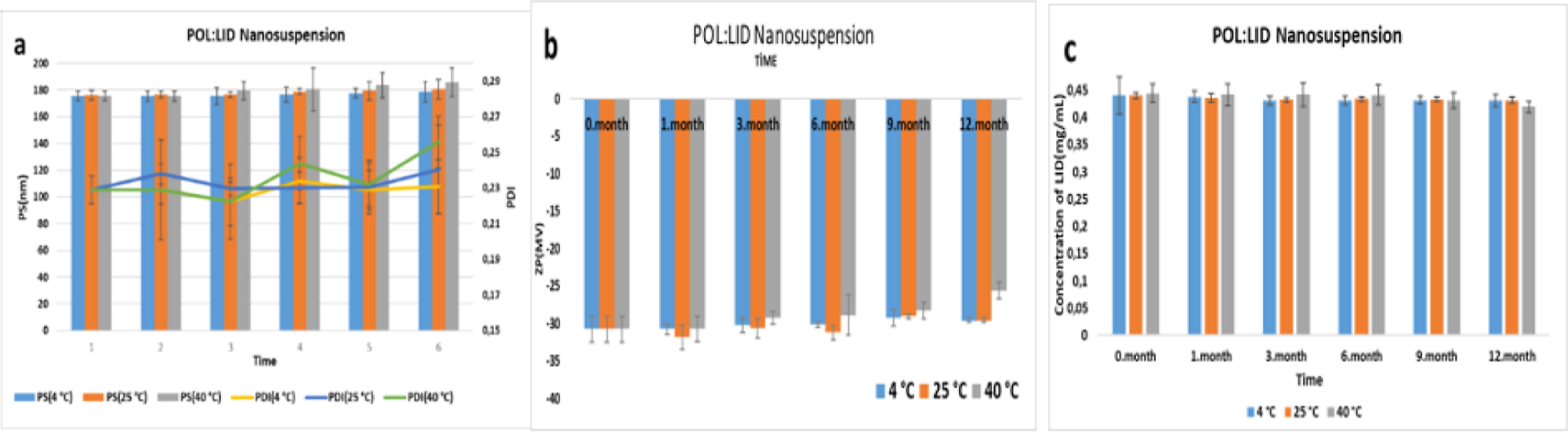
12 month (a,b) physical and (c) chemical results of POL/LID nanosuspensions.

Long-term stability results of the POL/LID nanosuspension are shown in Figure 14. It was observed that the PS, PDI, and ZP values of the POL nanosuspension did not change statistically for 12 months at 4 ± 2 and 25 ± 2 °C (*p* > 0.05). Similarly, Mishra et al. developed hesperetin nanosuspensions for dermal application. Poloxamer-stabilized nanosuspensions have been reported to be stable at room temperature.^77^

**Figure 15:**
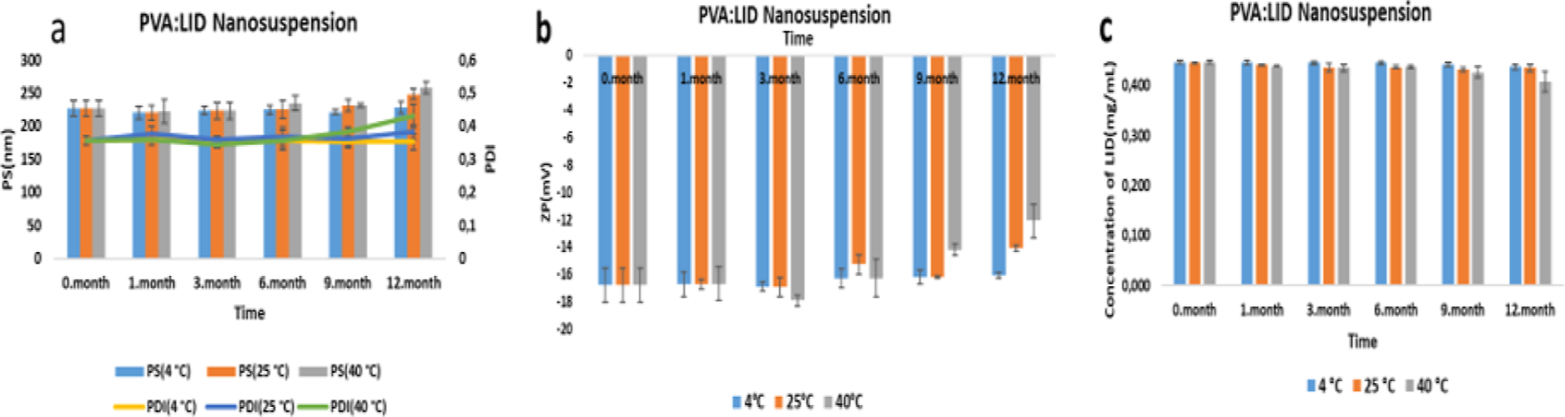
12 month (a,b) physical and (c) chemical results of PVA/LID nanosuspensions.

Stability results of PVA/LID nanosuspensions are listed in Figure 15. PS, PDI, and ZP results of PVA nanosuspensions up to 12 months at 4 ± 2 °C and up to 6 months at 25 ± 2 °C were found to be similar to the initial ones. As a result, it was observed that POL nanosuspension maintained its physical stability for 12 months at 4 ± 2 and 25 ± 2 °C, and PVA nanosuspension maintained its physical stability for up to 12 months at 4 ± 2 °C and up to 6 months at 25 ± 2 °C. Based on these data, it was concluded that the physical stability of nanosuspensions in which POL was used as a stabilizer was better.

### 3.13 In Vivo Studies

Tail flick testing was applied to determine analgesic and anesthetic effectiveness. This test is a frequently used test to detect the anesthetic effect. There are many studies in which the tail flick test was used to determine the anesthetic effectiveness of LID.^(^^78−81^^)^ Within the scope of this study, parameters such as latent time and analgesic effects are examined.

**Figure 16:**
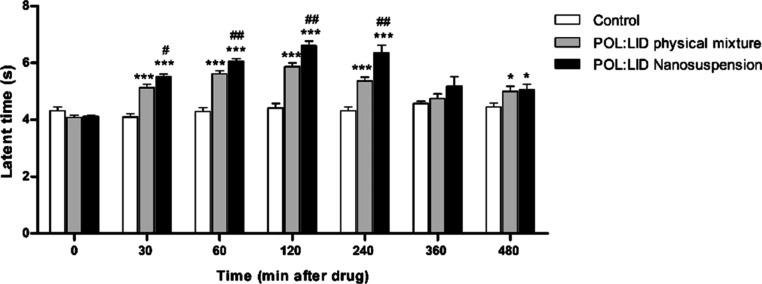
Effects of formulations on tail flick latency times [values were expressed as mean ± SEM (*n* = 6)]. Difference from control group: *p < 0.05, **p < 0.01, and ***p < 0.001. Difference from the POL/LID physical mixture group: #*p* < 0.05, ##*p* < 0.01, and ###*p* < 0.001).

The change in latent time of the formulations depending on time is given in Figure 16. Starting from the 30th minute, POL/LID nanosuspension significantly prolonged the tail flick time and showed a better analgesic-anesthetic effect than the control group and the POL/LID physical mixture (*p* < 0.05). In nanosuspensions, active substances barely soluble in water have a large-surface area, and therefore, both the dissolution rate and water solubility of the active substance increase. They provide active substance accumulation in the skin in nanoparticulate form, increasing skin penetration and bioavailability of active substance molecules by causing an increased concentration gradient.^5,6^ In addition, it has been reported that these systems increase the pharmacological effect of the active substance on the skin as it accumulates in hair follicles and increases the time it stays on the skin.^7^

**Figure 17:**
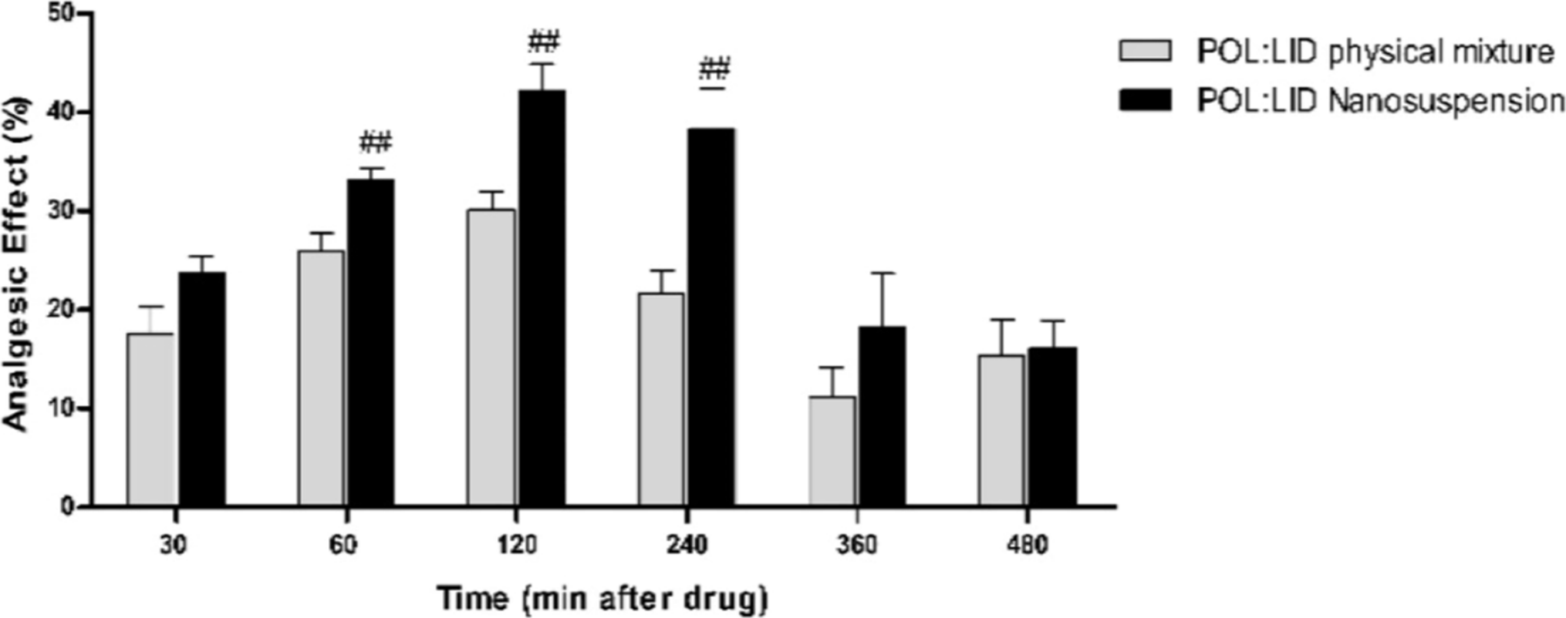
Analgesic effects of formulations [values were expressed as mean ± SEM (*n* = 6)]. Difference from the POL/LID physical mixture group: #*p* < 0.05, ##*p* < 0.01, and ###*p* < 0.001).

According to the data on the change of the analgesic effect over time (Figure 17), it was observed that the nanosuspension form of LID had a better anesthetic effect compared to its coarse form. It is seen that the detected analgesic effect reaches its maximum level at the 120th minute. It is also supported by the literature that nanosuspensions increase the effectiveness of the active substance compared to coarse suspensions.^35,82^

According to the literature review, evaluation is also made by calculating the area under the latent duration–time graph obtained from the tail-flick test.^(^^81,83,84^^)^ Shin et al. (2004) developed different LID gel formulations. They evaluated the anesthetic-analgesic effect using the area under the time-dependent tail flick graph (AUC) and tail flick time values.^81^ In our study, AUC values of the formulations were calculated based on latent period and % analgesic effect (Table 11). According to the data obtained, the LID nanosuspension showed a higher analgesic-anesthetic effect than the coarse suspension.

**Table 11:** AUC Values of Latent Time and % Analgesic Effect^a^

groups	latent time AUC values	% analgesic effect AUC values
**control**	100	100
POL-LID **physical mixture**	119 ± 2***	651 ± 128 ***
POL/LID **nanosuspension**	132 ± 3***, # #	893 ± 164 ***, # #

a (Values were calculated as the percentage of control and expressed as mean ± SEM (*n* = 6). Difference from control group: *p < 0.05, **p < 0.01, and ***p < 0.001. Difference from the POL/LID physical mixture group: #*p* < 0.05, ##*p* < 0.01, and ###*p* < 0.001).

## 4 Conclusions

LID nanosuspension formulations were successfully prepared by the wet milling method. Using the experimental design, nanosized stable nanosuspensions were prepared for both stabilizers (POL and PVA). DoE is a suitable approach for optimizing process parameters in the bead milling method. More stable and smaller particle-sized nanosuspensions were produced by using POL as a stabilizer. POL is an advantageous polymer in the production of nanosuspension formulations. Permeation of the active substance through the skin and release from the dialysis membrane were higher in nanosuspension formulations than that in coarse powder. In addition, thanks to nanosuspensions, the active substance accumulates more in the skin, increasing the local effect. Thus, the dermal bioavailability also increases. In this study, process parameters were optimized through experimental design in the production of nanosuspension formulations by the wet milling method. The effect of POL and PVA in nanosuspension formulation was investigated, and POL was found to be more advantageous. Additionally, in the in vivo study, it was concluded that nanosuspensions increased the analgesic/anesthetic effect compared to coarse suspensions.

## Data Availability

All data generated or analyzed during this study are included in this published article.
